# Two Cases of Paracentesis of the Thorax, for Empyema, with Favourable Results

**Published:** 1820-02

**Authors:** Dominique Novarra


FOR THE LONDON MEDICAL AND PHYSICAL JOURNAL.

### *Two Cases of Paracentesis of the Thorax, for Empyema, with favourable Results.* By Dr. Dominique Novarra.

A MAN, thirty-two years of age, suffered frequent cough, intense fever, difficulty of respiration, pain in the left side of the chest, and all the other symptoms of peripneumony. He was bled and purged twice without benefit. Being called to see the patient on the twentieth day of the disease, I found him with fever, a weak pulse, and a burning skin: he was distressed with ardent thirst; his tongue was coated; food seemed bitter to him, and excited nausea: respiration was laborious; and he had a harsh cough without expectoration. A dull pain was experienced in the left side, at an equal distance from the spine and sternum; and this pain became acute on the part being pressed by the finger. The left side of the chest appeared to be larger than the right.

From these symptoms, I had no doubt of the existence of a collection of purulent matter in the left side of the chest, and I proposed the operation of evacuating it; to which the patient refused to submit. Another practitioner, whom he consulted, gave him purgatives and other medicines, which did not relieve him. Violent drastics, administered by a rash empiric, produced enormous alvine evacuations, which brought him into a deplorable state. Tired, at length, at finding his hopes disappointed, he again had recourse to me. I found him very pale, having only the cheeks tinged with a little circumscribed redness; his eyes were sunken in their orbits, and his pulse was weak and febrile. On making pressure on the false ribs of the left side, a slight œdema was manifest there. The operation, to which he now readily submitted, was practised four months after the first invasion of the disease. At the instant the chest was opened, between the second and third false ribs, counting from above downwards, there sprung from the wound a full jet of purulent matter, the flow of which was accelerated by the respiratory motions; and the quantity evacuated was about twelve pints.* A seton was introduced into the wound, and it was covered by a light compress and bandage. The patient, having taken a composing potion, soon fell asleep. In the evening a glass-full more pus was passed from the wound. The night was tranquil; the difficulty of respiration had almost entirely disappeared. I ordered milk, rice, and veal-broth for his diet. Ten days after the operation, a little pus was passed by the wound. The pulse was very weak, but not febrile;

* There is a case published in a late number of the *Edinburgh Medical and Surgical Journal*, where an equal quantity of pus was evacuated under nearly similar circumstances, and with a favourable result.—Edit.

and, as the use of milk had caused diarrhœa, I ordered two grains of opium in an ounce of decoction of cinchona, which produced the desired effect. Twenty days after the operation, the patient rode on horseback, to the distance of a league and a half from his residence, without inconvenience. Two or three ounces of pus continued to flow daily, during a month, from this time. Soon after this the patient appeared in public, and had neither cough nor difficulty of respiration. He has now resumed his ordinary occupation, not experiencing any uneasiness, although a little pus still oozes from the wound.

---

FOR THE LONDON MEDICAL AND PHYSICAL JOURNAL.

